# Infestation of small ruminants by the metacestode stage of *Taenia hydatigena* in slaughterhouse, North East Tunisia

**DOI:** 10.1002/vms3.222

**Published:** 2019-11-29

**Authors:** Khouloud Khaled, Ghaleb Teber, Faten Bouaicha, Safa Amairia, Mourad Rekik, Mohamed Gharbi

**Affiliations:** ^1^ Laboratoire de Parasitologie Institution de la Recherche et de l’Enseignement Supérieur Agricoles & Univ. Manouba École Nationale de Médecine Vétérinaire de Sidi Thabet Sidi Thabet Tunisia; ^2^ Municipality of Menzel Temime Menzel Temime Tunisia; ^3^ International Center for Agricultural Research in the Dry Areas (ICARDA) Amman Jordan

**Keywords:** *Cysticercus tenuicollis*, Sheep, Slaughterhouse, Tunisia

## Abstract

**Background:**

*Cysticercus tenuicollis* (larvae of *Taenia hydatigena*) is a frequent cosmopolitan endoparasite of ruminants. The infestation by this parasite is underestimated since it is neither zoonotic nor inducing high economic losses in the sheep sector.

**Methods:**

This study aimed at estimating different parasitological indicators, *Cysticercus tenuicollis* infestation in small ruminants using a slaughterhouse‐based survey in Northeast Tunisia. A total number of 3,692 sheep and 78 goats were examined in the slaughterhouse of Menzel Temime.

**Results:**

The overall prevalence was estimated to be 2.8 (106/3692) and 8.9% (7/78) in sheep and goats, respectively. The abundance in these two species was 0.24 and 0.05 and the intensity 1.97 and 2.85, respectively. In goats, all the cysts were found in the mesentery, whilst, in sheep, the majority were in mesentery (96%) but also on the liver in 2% of the cases and in both organs (2%). There were only cysts with aqueous liquid with predominantly middle‐sized cysts (1 to 3 cm) corresponding to 63.15 and 70.34% in sheep and goats, respectively.

**Conclusion:**

Since, in small ruminants, the infestation by *C. tenuicollis* indicators are not high, low cost control measures should be implemented to eliminate this parasite in Northeast Tunisia.

## Graphical abstract

The authors estimated the infestation prevalence of sheep and goats by *Cysticercus tenuicollis*. The overall prevalence was estimated to 2.8 (106/3692) and 8.9% (7/78) in sheep and goats, respectively. The abundance in these two species was 0.24 and 0.05 and the intensity was 1.97 and 2.85, respectively.

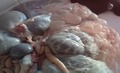

## 1 INTRODUCTION

Sheep face several parasitic infestations that are generally asymptomatic. Despite this cryptic feature of these infestations, they are important since they have high prevalence and are generally persistent in time and their number is high. This is due to a multiplication effect (high prevalence x high number of parasites x long term infections) inducing high losses that are underestimated. In Sardinia, the losses due *T. hydatigena* infection in sheep were estimated to be € 333,657.7/year (Scala et al., 2015). These authors considered only liver condemnation and proper disposal of these organs. The annual cost of liver condemnation due to *C. tenuicollis* was the lowest among other sheep parasites in Iran: hydatid cyst (US$ 8,655,154), *Dicrocoelium dendriticum* (US$8,099,418), *Fasciola* spp. (US$ 7,948,332) and *Cysticercus tenuicollis* (US$ 93,726) (Jahed Khaniki, Kia, & Raei, 2013).

*Taenia hydatigena* is a cosmopolitan cestode that infests the intestine mainly in dogs but also in several other carnivores (fox, weasels, stoats, polecats, wolfs, hyenas). The final hosts shed eggs in their faeces that contaminate other mammalian species. The metacestode stage of *Taenia hydatigena* (*Cysticercus tenuicollis* infestation) infests small ruminants and secondarily, cattle, goats, pigs, deers and horses (Taylor, Coop, & Wall, 2013). Other wild species, namely the taruca (*Hippocamelus antisensis*) and the red brocket deer (*Mazama americana*) were identified as intermediate hosts of *T. hydatigena* in Peru (Gomez‐Puerta, Pacheco, Gonzales‐Viera, Lopez‐Urbina, & Gonzalez, 2015). This parasite is not considered as an important health issue but in some cases, parasites’ migration induces hepatitis. High infestations lead in some cases to death. Scala et al. (2016) reported for example the death of five lambs out of 21 due to *C. tenuicollis* infestation. A morphological and biochemical study carried out in Tiart (western Algeria) suggested the presence of several sub‐species of *C. tenuicollis* infesting small ruminants. This could represent one of the determinants that influences the epidemiological pattern of this parasitic infestation (Kouidri et al., 2018). Senlik (2008) did not find statistically significant differences in the infestation prevalence according to breed (Kivircik and Merino sheep breeds), sex and age. Nevertheless, the same author showed that, infestation intensity was significantly higher in Kivircik compared to Merino sheep and in males compared to females.

The aim of this study is to estimate different epidemiological infestation indicators of slaughtered sheep and goats at the local slaughterhouse of Menzel Temime (Northeast Tunisia).

## 2 MATERIALS AND METHODS

### 2.1 Study region and animals

**Figure 1:**
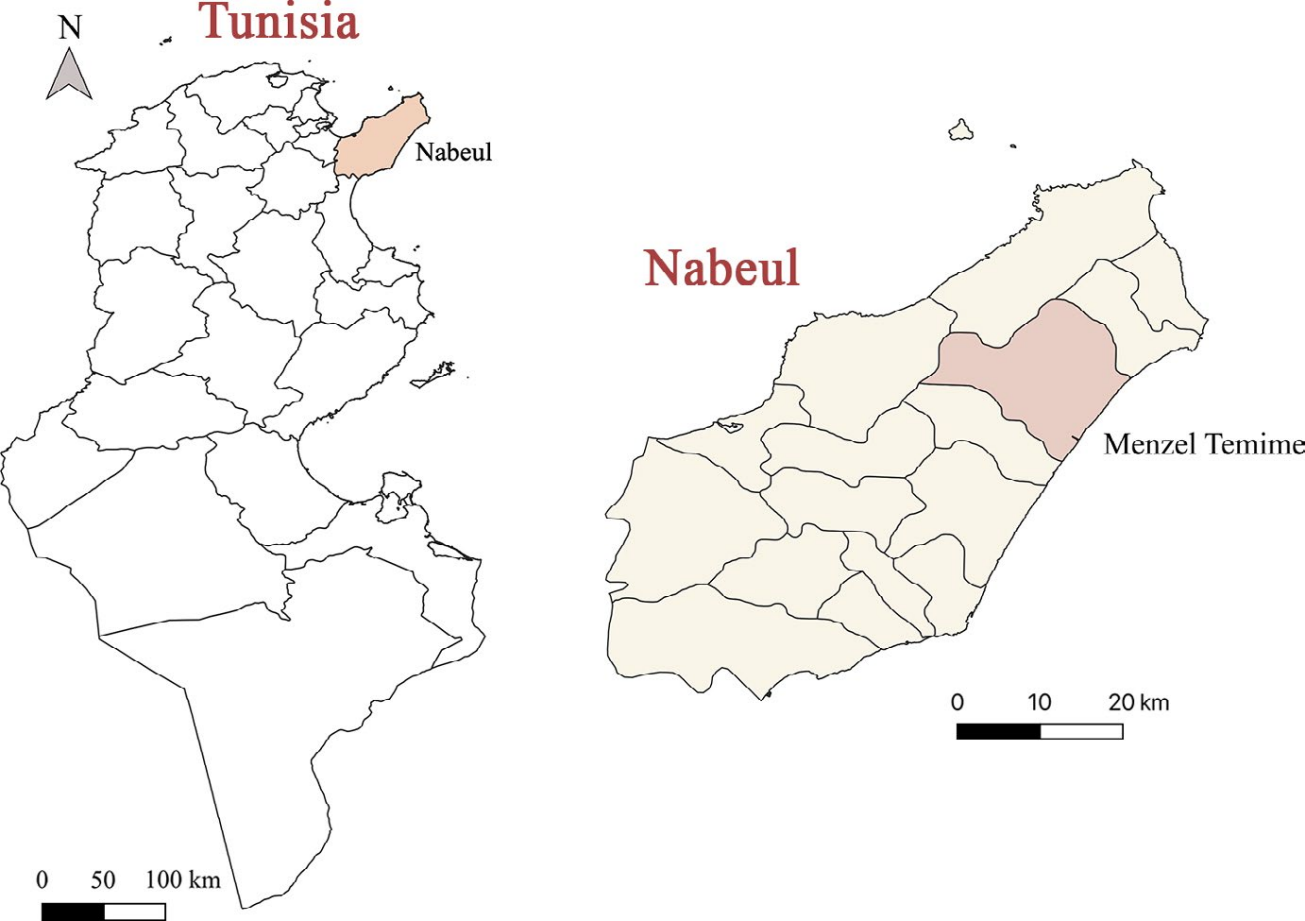
Geographic localization of Menzel Temime locality (North East Tunisia) (QGIS ©)

The present survey was carried out in the locality of Menzel Temime, Northeast Tunisia (Figure 1) totalling a population of 60,000 sheep heads. The climate of this region is classified as BSh in the Köppen and Geiger classification; with a mean annual rainfall of 252 mm. August (26.8°C) and January (10.5°C) are the hottest and coldest months, respectively (Open Street Map project 2015).

Between January and March 2018, a total number of 3,692 sheep and 78 goats were examined at the local slaughterhouse of Menzel Temime (Table 1). The animals were mainly from the locality of Menzel Temime but a small number of animals came from other regions of Tunisia. The sex of each animal was recorded and animals were randomly ranked into three age groups < 3 years, 3 ≤ <6 years and ≥6 years.

**Table 1:** Epidemiological indicators of sheep and goats’ infestation by *Cysticercus tenuicollis* cysts

Epidemiological indicator	Item	Infested/examined (%)	
		Sheep	Goats
Prevalence	*Sex*		
	Males	40/1739 (2.3)	3/46 (6.5)
	Females	66/1953 (3.4)	4/32 (12.5)
	*Age*		
	<3 years	86/3133 (2.7)	7/78 (8.9)
	3 ≤ <6 years	5/35 (14.3)	0
	≥ 6 years	15/524 (2.9)	0
	*Month*		
	January	10/1210 (0.8)	0/23
	February	32/1284 (2.5)	1/22 (4.5)
	March	64/1198 (5.3)	6/33 (18.2)
	*Overall*	106/3692 (2.8)	7/78 (8.9)
Abundance	*Sex*		
	Males	90/1739 (0.05)	16/46 (0.34)
	Females	119/1953 (0.06)	3/32 (0.09)
	*Age*		
	<3 years	166/3133 (0.05)	19/78 (0.24)
	3 ≤ <6 years	8/32 (0.22)	0
	≥ 6 years	35/527 (0.06)	0
	*Month*		
	January	27/1210 (0.02)	0/23
	February	72/1284 (0.05)	1/22 (0.04)
	March	110/1198 (0.09)	18/33 (0.54)
	*Overall*	209/3692 (0.05)	19/78 (0.24)
Intensity	*Sex*		
	Males	90/40 (2.25)	16/3
	Females	119/66 (1.8)	3/4
	*Age*		
	<3 years	166/86 (1.93)	19/7
	3 ≤ <6 years	8/5	0
	≥6 years	35/15	0
	*Month*		
	January	27/10	0
	February	72/32 (2.25)	1/1
	March	110/64 (1.71)	18/6
	*Overall*	209/106 (1.97)	19/7

### 2.2 Parasites’ collection

After being slaughtered, all the animals were screened for the presence of *C. tenuicollis* vesicles in the abdominal cavity and organs. For the infested animals, the localization of the cysts and their aspect were registered. The size of all the vesicles were measured and they were ranked into: small (<1 cm), middle sized (1 ≤ 3 cm) and big (<3 cm). All the vesicles were stored in identified vials containing 70° ethanol.

### 2.3 Estimation of parasitological indicators and statistical analysis

Three parasitological indicators were estimated as follows (Bush, Lafferty, Lotz, & Shostak, 1997):

Prevalence (%) = 100 x number of infested animals/ number of examined animals.

Abundance = number of parasites/ number of examined animals.

Intensity = number of parasites/ number of infested animals.

The percentages were compared using the Chi‐square Mantel‐Haenszel test and the means were compared using *Student t* test. All tests were performed at 5% threshold (Schwartz, 1993).

## 3 RESULTS

### 3.1 Infestation prevalence, abundance and intensity

The overall infestation prevalences by *Cysticercus tenuicollis* were 2.8 (106/3692) and 8.9% (7/78) in sheep and goats, respectively (*p* = .1) (Table 1).

### 3.2 Frequency of infestation intensity

**Figure 2:**
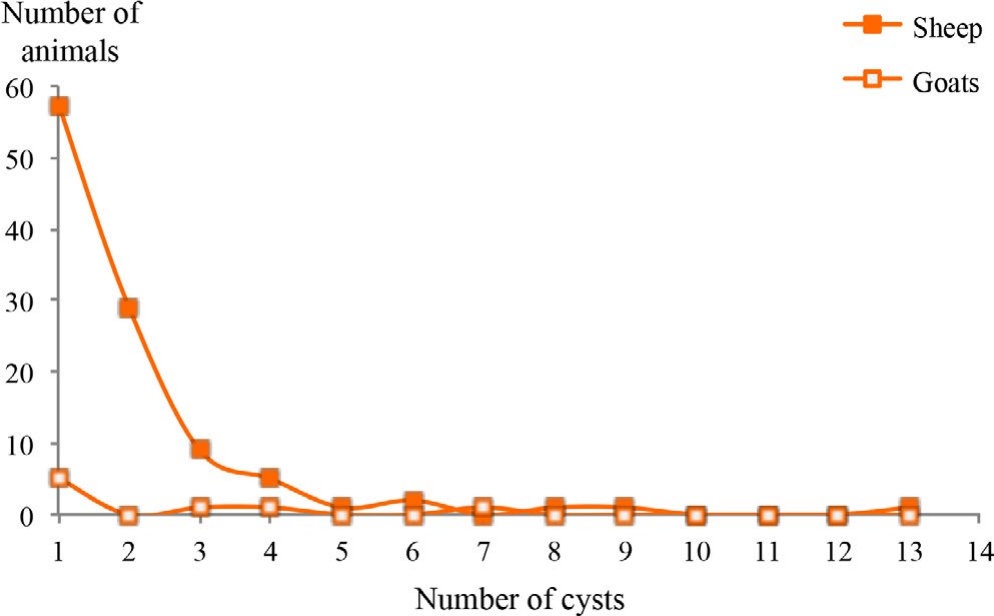
Frequency of different infestation intensities in sheep and goats

The maximum number of cysts was 13 but more than half (53.8%) of the sheep were infested by only one cyst and 94.3% of the sheep had four cysts or less (Figure 2).

### 3.3 Characteristics of *Cysticercus tenuicollis* cysts

All the cysts were either small or medium‐sized; in the majority of sheep (70.3%; 147/209) and goats (63.2%; 12/19), nearly 70% of the cysts (159/228) were of medium size, between 1 and 3 cm.

In goats, all the cysts were found in the mesentery, whilst, in sheep, the majority were in mesentery (96%) but also found on the liver only (2%) and in both organs (2%). All the collected cysts contained liquid and none was either calcified or degenerated.

## 4 DISCUSSION

Among the examined sheep (*N* = 3,692) and goats (*N* = 78), 106 (2.8%) and 7 (8.9%) sheep and goats were infested, respectively. Working in the same region, Maamouri (2005), reported a similar trend with a higher infestation prevalence in goats (17.3%). In Tunisia, goats are kept by small‐scale poor farmers with a very limited veterinary health care leading to a higher infestation risk in this species when compared to sheep. Brahmi (2007) reported in Sidi Thabet region (North Tunisia) a significantly higher prevalence in both slaughtered sheep and goats, 63.89 and 84.23%, respectively. This could be due to a much higher concentration of sheep and shepherded dog populations in the studied region.

The infestation prevalence by this parasite is high in other regions of the world; Pathak and Gaur (1982), in India, reported an infestation prevalence of 37.03 and 27.29% in sheep and goats, respectively. Similar trends were reported in Ethiopia, where Sissay, Uggla, and Waller (2008) reported a prevalence of 79 and 53% in sheep and goats, respectively.

In the present study, the infestation prevalence in female sheep was significantly higher than in males (3.37 and 2.3%, respectively) (*p* < .05). In contrast, no such difference was found between male and female goats which may be explained by the small sample size. On the other hand the presence of a higher infestation prevalence in female sheep could be explained by the longer pasturing time spent by females since males are usually kept in the barns and mainly receive concentrate (with very little grass) as part of fattening practices. Infestation prevalence in young animals (<3 years) was 2.7%, it increases to 14.3% (3≤ <6 years old) then decreases to 2,9% (≥6 years) (*p* < .05). The decrease in infestation prevalence with age could be explained by the installation of a progressive efficient immunity in adult animals inducing the elimination of old cysts.

Maamouri (2005) reported a prevalence in lambs of 10.43%, but this value was roughly constant at different age classes (10.63% at 2 years, 11.49% at 3 years, 13.04% at 4 years and 13.51% at 5 years).

In Central Tunisia, Lahouar (2003) estimated the prevalence to 19.32% in lambs, 16.66% in 2‐year‐old, 12.5% in 3‐year‐old, 10% in 4‐year‐old and 5.33% in sheep of 5 years and more.

In Jordan, Torgerson, Williams, and Abo‐Shehada (1998) showed that the infestation prevalence did not increase with age in both sheep and goats. Indeed, *C. tenuicollis* is very immunogenic inducing the establishment of an effective immune response in both ruminant species. In the present study, the infestation intensity was almost constant in sheep according to age. Cabrera et al. (1995) reported no significant difference in infestation intensity in sheep according to age.

A similar trend was also observed in goats in Tunisia; infestation intensity was roughly constant according to age (1.72), it varied between 1 and 3.42 in sheep (mean 2.3) (Lahouar, 2003). The low infestation intensity could be explained by both the presence of an efficient immunity reducing the number of parasites but also by the low prevalence of the parasite in dogs.

During the study period (from January to March), the infestation prevalence varied in sheep from 0.82% to 5.34% (*p* < .05). The prevalence of cysts in UK decreased from 11% to 4% between April and July, respectively (Alaa, 2014). This variation could be explained by the proportion of receptive animals in the population and by the higher grazing intensity in April which exposes naïve animals to eggs shed by dogs.

In goats, cysts were present only in the mesentery; in sheep, the same trend was observed (96%) but 2% of the cysts were on the liver and 2% on the liver and the mesentery (*p* = .02). Lahouar (2003) found that in sheep, mean‐sized cysts were dominant (46%) but small cysts were dominant in goats (54%).

The aspect of the cysts is very important since it allows veterinarians to easily identify in the slaughterhouse any atypical cyst and differentiate it from other gross lesions, mainly zoonotic ones, i.e. tuberculosis and echinococcosis particularly when the cysts are degenerated. Tuberculosis is extremely rare in small ruminants since they are resistant; only sheep living in highly infected cattle flocks develop it (Gelalcha, Zewude, & Ameni, 2019). Gross lesions in small ruminants are in most of the cases generalized. *Echinococcus granulosus* cysts have different sizes with a liquid under pressure and a double membrane; they are localized in different organs but mostly in the lungs and the liver (Lahmar, Kilani, Torgerson, & Gemmell, 1999).

In the present study, only liquid cysts were collected. Maamouri (2005) reported that in goats, 78.6% of the cysts were liquid, whilst 17.3% were calcified and 4.3% were caseified. Lahouar (2003) found that cysts containing liquids were the most frequent in all age classes, representing 82.05 and 81.25% in sheep and goats, respectively.

Calcified cysts represented 14.52 and 14.5% of the total number of cysts in sheep and goats, respectively. Whilst, calcified cysts were present in only 3.41 and 4.16% in sheep and goats, respectively. The presence of exclusively viable cysts in the present study could be explained by the young age of the majority of the examined animals.

Infestation by *T. hydatigena* cysts is frequently unapparent with no visible impact on the health status of infested animals. Nevertheless, these parasites induce liver lesions with a small weight loss but in some cases, this infestation is lethal. The field veterinarian could easily identify these parasites even in their atypical forms. As the infestation prevalence is low in Northeast region of Tunisia, a control program could be implemented in order to eliminate this parasite with low costs and efforts.

## CONFLICT OF INTEREST

The authors declare that they have no competing interests.

## ETHICAL STATEMENT

This study was conducted in accordance with relevant national and international guidelines on handling animals, taking care to respect animal welfare.
